# Circadian rhythms in colonic function

**DOI:** 10.3389/fphys.2023.1239278

**Published:** 2023-08-30

**Authors:** Timothy J. Hibberd, Stewart Ramsay, Phaedra Spencer-Merris, Phil G. Dinning, Vladimir P. Zagorodnyuk, Nick J. Spencer

**Affiliations:** ^1^ College of Medicine and Public Health, Flinders University, Adelaide, SA, Australia; ^2^ Colorectal Surgical Unit, Division of Surgery, Flinders Medical Centre, Adelaide, SA, Australia

**Keywords:** colon, circadian rhythms, colonic motility, enteric nervous system, time of day, pain signaling, colonic absorption, colonic manometry

## Abstract

A rhythmic expression of clock genes occurs within the cells of multiple organs and tissues throughout the body, termed “peripheral clocks.” Peripheral clocks are subject to entrainment by a multitude of factors, many of which are directly or indirectly controlled by the light-entrainable clock located in the suprachiasmatic nucleus of the hypothalamus. Peripheral clocks occur in the gastrointestinal tract, notably the epithelia whose functions include regulation of absorption, permeability, and secretion of hormones; and in the myenteric plexus, which is the intrinsic neural network principally responsible for the coordination of muscular activity in the gut. This review focuses on the physiological circadian variation of major colonic functions and their entraining mechanisms, including colonic motility, absorption, hormone secretion, permeability, and pain signalling. Pathophysiological states such as irritable bowel syndrome and ulcerative colitis and their interactions with circadian rhythmicity are also described. Finally, the classic circadian hormone melatonin is discussed, which is expressed in the gut in greater quantities than the pineal gland, and whose exogenous use has been of therapeutic interest in treating colonic pathophysiological states, including those exacerbated by chronic circadian disruption.

## Introduction

Biological rhythms that persist on a roughly 24-h cycle under stable environmental conditions, can be synchronized by external cues (zeitgebers), and retain constancy across varying physiological temperatures are classified as circadian (Aschoff, 1981). However, most studies on daily rhythms in colonic function do not rigorously test these criteria, which makes their findings suggestive but not definitive of circadian rhythmicity. In this review, such instances will be categorized under “daily rhythms,” while evidence meeting circadian criteria will be explicitly identified. In discussing genes and proteins, the review primarily draws on mouse data, using “*Clock*” for gene or messenger RNA, and “CLOCK” for the protein.

Circadian rhythms are present in mammals, tuning cell and organ processes to the ambient 24-h light-dark cycle, optimising and coordinating bodily functions including feeding (Segers and Depoortere, 2021), defecation (Duboc et al., 2020), and urination (Noh et al., 2011). Controlling the body’s rhythmicity is a hierarchical system comprised of multiple functionally overlapping circadian oscillators. At the top of the hierarchy is the main light-entrainable clock of the circadian system, which lies within the suprachiasmatic nucleus (SCN) of the hypothalamus containing around 20,000 neurons (Ralph et al., 1990; Hastings et al., 2018; Yan et al., 2020). A ∼ 24-h circadian cycle must be reset by a daily cue (zeitgeber) to be synchronized with external environmental time (Duffy and Czeisler, 2009). Light is the primary zeitgeber for the SCN. In mammals, the SCN is principally entrained by retinal melanopsin-expressing non-visual photoreceptors (intrinsically-photosensitive retinal ganglion cells) that detect the light environment (Schlangen and Price, 2021) and signal via the retinohypothalamic tract. The SCN signals to other parts of the brain via projections to *local circadian clocks of the brain centres* that control cognition, mood, behavioural rhythms such as sleep-wakefulness and feeding-fasting, and autonomic and neuroendocrine circadian rhythms (Hastings et al., 2018).

Cell rhythmicity in the SCN involves a core molecular oscillator referred to as the transcription-translation feedback loop (TTFL). See Table 1 for the expanded names of TTFL components. The TTFL may be considered an interaction between positive transactivating elements through CLOCK/BMAL1 and negative transinhibiting elements through PER/CRY (Lowrey and Takahashi, 2011). The core mammalian TTFL pacemaking loop involves nuclear transcription of the *Clock* and *Bmal1* genes, followed by post translational cytosolic heterodimer formation of CLOCK-BMAL1 protein complexes (Reppert and Weaver, 2002). Succeeding nuclear translocation of CLOCK-BMAL1 drives daytime expression of *Per1/2* and *Cry1/2* through E box enhancers. The formation and increasing levels of subsequent PER-CRY protein complexes (with Ck1δ; Cao et al., 2023) inhibit *Per* and *Cry* expression via CLOCK-BMAL1 E box dissociation (Hastings et al., 2018; Cao et al., 2021), possibly driving CLOCK-BMAL1 to act at other DNA sites (Koch et al., 2022). A decrease in *Per* and *Cry* mRNA levels and proteasomal degradation of PER-CRY complexes (Hastings et al., 2018) lead to a disinhibition that enables the next CLOCK/BMAL1-driven cycle (Lowrey and Takahashi, 2011). Genomic and proteomic regulation of *Per* and *Cry* takes ∼24 h. In mouse SCN, PER shows large circadian fluctuations in abundance (Yamaguchi et al., 2003), whilst BMAL1, CLOCK and CRY protein levels are more constantly expressed showing lower amplitude circadian rhythmicity (von Gall et al., 2003; Maywood et al., 2013; Yang et al., 2020). The core loop comprising BMAL1-CLOCK and PER-CRY drives ancillary, interlocking TTFLs through proteins ROR*α/β*, and REV-ERB*α/β* that stabilize the core loop period and amplitude (Cho et al., 2012), and through DBP and NFIL3 (Takahashi, 2017). Together these transcription factors also drive rhythmic expression of other genes via their respective promotors (i.e., clock-controlled genes outside the TTFL), thus coupling the molecular oscillator to cell functions (Takahashi, 2017).

**TABLE 1:** Gene, protein, and expanded names of components of the transcription-translation feedback loop.

*Clock*, CLOCK	Circadian locomotor output cycles kaput
*Bmal1*, BMAL1	brain and muscle ARNT (aryl hydrocarbon receptor nuclear translocator)-like protein 1 (also known as Mop3)
*Per1/2*, PER1/2	period 1, period 2
*Cry1/2*, CRY1/2	cryptochrome 1, cryptochrome 2
*Csnk1d*, CK1 δ	casein kinase 1 delta
*Rorα/β*, RORα/β	retinoic acid receptor-related orphan receptor alpha/beta
*Rev-erbα/β*, REV-ERBα/β	reverse-erythroblastosis virus alpha/beta (also known as NR1D1/NR1D2)
*Dbp*, DBP	D site albumin promoter binding protein
*Nfil3*, NFIL3	nuclear factor, interleukin 3, regulated

Remarkably, the core TTFL also operates in the cells of peripheral tissues and organs (termed “*peripheral clocks*”) such as in the gut, liver, bladder, adipose tissue and skeletal muscle (Labrecque and Cermakian, 2015; Reinke and Asher, 2016; Basinou et al., 2017; Hastings et al., 2018). Thus, the same molecular oscillator underlies the rhythmic output of vastly different gene sets, depending on the tissue/cell type (Partch et al., 2014). The cell specificity of oscillator controlled outputs is achieved in part by components of the TTFL binding other transcription factors and nuclear receptors to suppress or enhance a cell specific transcription program (Patke et al., 2020). In addition, the output of the molecular oscillator can be differentiated by variations in genome and chromatin access in a cell/tissue specific manner (Patke et al., 2020). It is worth noting that core clock proteins interact with histone acetyltransferases to induce chromatin states that allow transcription to take place and that this process involves regulation by the histone deacetylase, SIRT1; a protein sensor of energy status (Takahashi, 2017). This contributes to a mechanism by which feeding behaviour and diet composition can modify the molecular oscillator (for review, see Sato and Sassone-Corsi, 2022).

**FIGURE 1:**
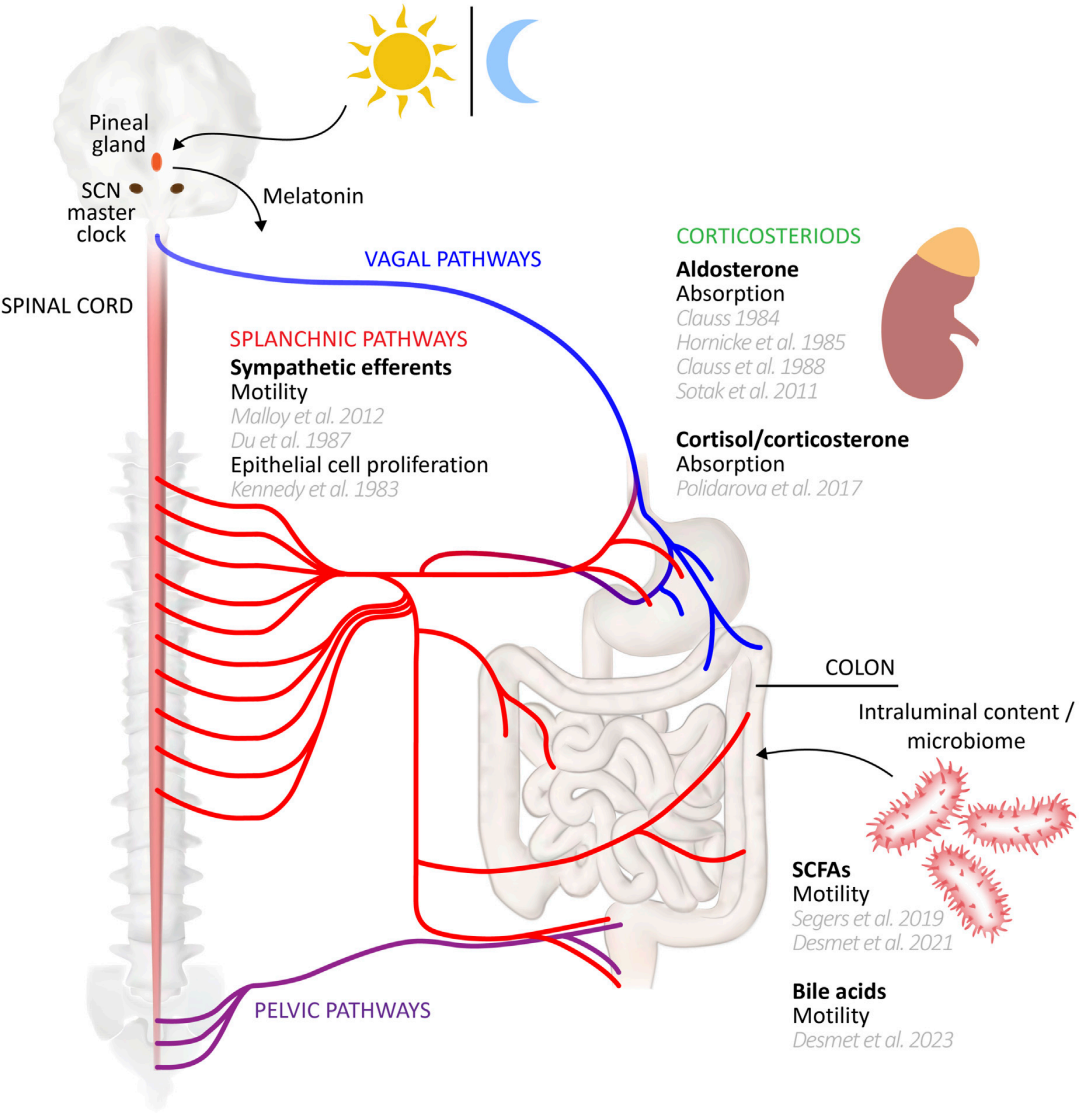
Circadian entrainers of colon function. A range of influences may entrain or modulate peripheral clocks underlying circadian rhythms of colonic functions. This schematic diagram summarizes those influences, citing supporting studies. Several influences, such as the vagal and pelvic efferent and afferent pathways remain to be studied in detail for their potential role in driving colonic function rhythmicity. Gut-CNS schematic based on Young (2012).

Peripheral clocks drive rhythmic expression of different gene sets in a cell specific manner. In addition, where identical non-clock genes are rhythmically expressed in different organs/cell types of the mouse, their peak expression timing nevertheless differed in phase by many hours, or indeed were antiphase (Zhang et al., 2014). Yet core clock gene phases were more aligned, each peaking within a window of ∼3 h across multiple tissues, indicating significant divergence in regulation of the non-clock genes between cell types (Zhang et al., 2014). The acrophase of *Bmal1* in mouse stomach and colon was similarly within 3 h of the SCN, but *Per2* diverged by up to ∼10 h (Hoogerwerf et al., 2007). The question thus arises as to the mechanisms coupling/entraining and maintaining phase relationships between central and peripheral clocks (for review, including intercellular coupling within tissues, see Astiz et al., 2019 ; Finger et al., 2020 ; Pilorz et al., 2020). In the case of the colon, the major candidate links to the SCN include neural inputs from the parasympathetic and sympathetic divisions of the autonomic nervous system, circulating hormonal factors, and the rhythmicity of feeding behaviours (see schematic diagram, Figure 1). Evidence for the roles of these mechanisms in maintaining rhythmicity of colonic functions and clock gene expression is discussed throughout this review. More generally, the SCN clock regulates the oscillation of peripheral clocks directly by neural signalling through sympathetic and parasympathetic nerves, and hormonal signalling via pineal and adrenal glands (Dickmeis, 2009; Ohdo, 2010; Richards and Gumz, 2012; Astiz et al., 2019), and indirectly through its influence on behaviours like sleep-wake cycles and feeding (Dibner et al., 2010). For the gut and liver peripheral clocks, one of the most important SCN-driven mechanisms is the temporal control of feeding, since food intake is a significant entraining cue (Damiola et al., 2000; Stephan, 2001; Stokkan et al., 2001; Stephan, 2002). Food intake entrains the circadian rhythm of clock genes in the gut, while those in the liver may be entrained via insulin secretion which subsequently regulates *Per1/2* expression (Finger et al., 2020; Zhang et al., 2020; Taleb and Karpowicz, 2022). Inversion of feeding times in mice results in an inversion of peripheral clocks in the gut, but not the SCN (Hoogerwerf et al., 2007). Indeed, peripheral and local oscillators outside the SCN that can control general activity rhythms are implied by experiments showing that non-photic cues such as timed food access (the food-entrainable oscillator; FEO) and methamphetamine administration (methamphetamine sensitive circadian oscillator; MASCO) can restore rhythmicity after SCN disruption, but little is known of their anatomical substrates (Mistlberger, 1994; Menaker et al., 2013; Pendergast and Yamazaki, 2018; Mistlberger, 2020; Taufique et al., 2022).

As seen in most mammals, including humans, both faecal defecation and urinary voiding exhibit a daily rhythm of increased occurrence during the “active period” (i.e., the daytime in diurnal animals, and night in nocturnal animals) and a decreased occurrence during the “inactive period,” or “rest period” (Kirkland et al., 1983; Herrera and Meredith, 2010; Noh et al., 2011; Negoro et al., 2012; Duboc et al., 2020). In humans, faecal defecation peaks early morning, usually shortly after waking, and following a meal (Heaton et al., 1992). Similarly, urinary voiding also peaks during the early morning, with a consistent pattern throughout the day and little to no occurrence at night (Noh et al., 2011). Chronic disruption to circadian rhythms can significantly impact health, sleep and quality of life (Xie et al., 2019; Vetter, 2020), with recent research turning towards the hormone melatonin as one of the potential treatments.

The SCN drives the activation of sympathetic nerves in the superior cervical ganglia that project to the pineal gland, evoking nocturnal melatonin synthesis and release into the circulation (Reiter, 1991; Claustrat et al., 2005). Melatonin could be partially responsible for synchronisation of the peripheral clocks by the central clock, but also serves as a feedback mechanism to the SCN (Prasai et al., 2011). Plasma levels of melatonin represent one of the most robust circadian rhythms with concentrations in the blood and urine peaking during the night, stabilising the sleep-wake cycle (Reiter et al., 2009). In the SCN, melatonin acts via G-protein coupled receptors; melatonin 1 (MT1) receptors reducing neuronal activity, and melatonin 2 (MT2) receptors causing a circadian phase shift (Dubocovich, 2007). MT1 and MT2 receptors have been identified in the neurons of the central nervous system (CNS) and peripheral organs such as blood vessels, heart, lung, kidney, bladder, liver, gut, and others (Dubocovich and Markowska, 2005; Pandi-Perumal et al., 2008). Exogenous melatonin can act peripherally on smooth muscle and enteric neurons influencing colonic motility, albeit in concentration ranges significantly higher than its physiological levels. Symptoms of functional dyspepsia, irritable bowel syndrome (IBS) and ulcerative colitis (UC) are significantly exacerbated by circadian disruptions (Kim et al., 2013; Fowler et al., 2022). Melatonin has been considered a potential treatment for gut and bladder disorders, such as functional dyspepsia, IBS (Lu et al., 2009; Chojnacki et al., 2013; Fowler et al., 2022), UC (Liu and Wang, 2019), and nocturia (Drake et al., 2004; Ramsay and Zagorodnyuk, 2023). This review summarises the circadian rhythmicity of the colon and the influence of melatonin on its function.

### Circadian rhythms of colonic motility

The large intestine receives from the ileum undigested content as well as endogenous secretions, metabolites and dead epithelial cells. Undigested material may be fermented by microbiota in the caecum and proximal colon. In the more proximal regions, intraluminal content is an amorphous semi-liquid. Water, electrolytes, and microbial products are absorbed along the colon as the content forms a stool that is released on defecation (Costa et al., 2021). These processes, including the motor behaviours that propel content along the large intestine show distinct circadian profiles.

#### Defecation

Defection is an overt indication of colonic motility that shows daily rhythmicity, peaking in the active period. This has been reported in numerous species, including diurnal humans (Rendtorff and Kashgarian, 1967; Heaton et al., 1992; Aschoff, 1994; Shemerovskii, 2002) and non-human primates (Bernstein, 1964; Caton et al., 1996), birds (Clarke, 1979; Rodriguez-Sinovas et al., 1994; Malek et al., 2020), dogs (Hirabayashi et al., 2009), horses (Piccione et al., 2005), camels (Aubè et al., 2017), and sheep (Piccione et al., 2005); and nocturnal rodents (Gosling, 1979; Magot and Chevallier, 1983; Firpo et al., 2005; Hoogerwerf et al., 2010; de Azevedo et al., 2011; Platt et al., 2013; Allen and Johnson, 2018), foxes (Klenk, 1971), antechinus (Cowan et al., 1974), rabbit (Jilge, 1974; Jilge and Hudson, 2001), hare (Pehrson, 1983), and house musk (Kobayashi et al., 2022). Some species, such as degu and the Mongolian gerbil that can show either diurnal or nocturnal activity patterns (Refinetti, 2006) have a more constant defecation pattern (Kenagy et al., 1999). Animals showing activity and defecation peaks around the day-night transitions may be referred to as “crepuscular,” such as the predominantly nocturnal cat (Wienbeck and Kreuzpaintner, 1976) and diurnal guinea pig (Elfers et al., 2021).

Most observations of the daily rhythmicity in defecation patterns arise from subjects with typical, ongoing photoperiods and *ad-libitum* food access. However, the persistence of defecation patterns during the active period under constant lighting conditions has also been identified in mice (Hoogerwerf et al., 2010), rabbits (Jilge, 1982) and humans (Aschoff, 1994). This suggests daily rhythms in defecation is not acutely sensitive to lighting conditions and thus likely represents an endogenous circadian rhythm. Yet, daily feeding rhythms show circadian rhythmicity and food intake potently stimulates gut motility, including defecation (Dorfman et al., 2022). Thus it remains possible that defecation patterns are not intrinsically circadian but is triggered by processes that are, such as feeding. This is tricky since food ultimately supplies most colonic content so its restriction limits defecation capacity. Interestingly however, restricting food availability to a 4-h period in rabbits during the light (inactive) period fully shifted hard faeces defecation to this period, along with general activity patterns (Jilge and Stähle, 1993). This illustrates the potency of the FEO in this species and the importance of food intake and availability in determining defecation and activity patterns. Whilst these data point to the potential role of a different oscillator and/or zeitgeber in determining daily rhythmicity of defecation, it does not clarify whether defecation patterns reflect intrinsic circadian rhythmicity of the colon. In a more recent study, the food intake and fecal pellet output of guinea pigs was tracked hourly, under normal light/dark conditions and *ad-libitum* food access (Elfers et al., 2021). An interesting finding of this study was that although guinea pigs consumed less food during the dark (inactive) period, the difference was modest, and the animals continued to consume food at around 65% of the mean active period rate. At the same time, mean fecal pellet output fell to near zero for most of the inactive period, and overall was less than 20% of the active period rate (Elfers et al., 2021). This would suggest daily defecation patterns are governed by more factors than food intake alone, pointing to the possibility of true intrinsic circadian rhythmicity of colonic motor behaviours.

#### Colonic motor behaviours

The motor behaviours of the entire gastrointestinal tract are under circadian influence (for review, see Leembruggen et al., 2022). Here we principally focus on colonic motor behaviours and adjacent regions. Most studies that describe daily variability in colonic motor activity, *in vivo*, has been done in humans in 24-h manometry studies (Bassotti et al., 1999; Bharucha and Brookes, 2012). One of the most prominent motor activities of the human colon are referred to as high amplitude propagating contractions (HAPCs). HAPCs are strong propulsive contractions that typically initiate in the proximal colon and may mediate defecation (Corsetti et al., 2019). Compatible with circadian rhythmicity of human defecation, human colonic manometry studies report most (up to 90%) HAPCs occur in the daytime and are relatively rare at night (Narducci et al., 1987; Bassotti and Gaburri, 1988; Crowell et al., 1991; Bassotti et al., 1992; Furukawa et al., 1994; Hagger et al., 2002; Rao et al., 2010). Where studies report the hourly distribution of HAPCs, the peak occurrence has been detected at awakening ∼7a.m. (Bassotti and Gaburri, 1988; Bassotti et al., 1992), just after breakfast ∼9a.m. (∼7a.m. wake, 8a.m. breakfast) (Narducci et al., 1987) and following a 12p.m. lunch at ∼1p.m. (Crowell et al., 1991). The preponderance of HAPCs in the day (active) period was observed where subjects were confined to a supine or side-lying position for recordings, indicating ambulation cannot fully account for daily HAPC variability (Narducci et al., 1987; Bassotti and Gaburri, 1988; Bassotti et al., 1992; Furukawa et al., 1994). Food intake is a well-known stimulus of HAPCs and other colonic motor patterns, taking effect within minutes of eating and lasting up to 2 h postprandially (Dinning et al., 2014). The rate of HAPCs increases just prior to, or upon waking in the morning, before breakfast (Crowell et al., 1991; Bassotti et al., 1992; Furukawa et al., 1994). This suggests daily rhythmicity of HAPCs is not fully accountable by a simple response to feeding, and thus more likely to be circadian.

HAPCs may be important for colonic propulsion but represent a small proportion of the motor patterns present in the human colon. Several lower amplitude propagating motor patterns have been identified by high resolution manometry. The most prominent of these is the cyclic motor pattern. This motor pattern consists of rhythmic pressure waves, occurring between 2-6 cycles per minute, that can propagate in an antegrade or retrograde direction. Single propagating contractions of varying length, speed and polarity can also occur (Dinning et al., 2014; Dinning et al., 2016). Given the short duration of colonic high-resolution manometry studies (typically between 4-8hrs), the daily rhythmicity of motor patterns quantified with this technique has not been established. However, in low-resolution manometry studies the aggregate area under the curve and frequency of all ongoing contractility (not just HAPCs) along the human colon was significantly suppressed at night compared to the day (Narducci et al., 1987; Soffer et al., 1989; Furukawa et al., 1994; Hagger et al., 2002; Rao et al., 2004; Rao et al., 2010). Furthermore, low-resolution manometry studies had identified bouts of rhythmic contraction in the rectum with the same frequency as the cyclic motor pattern described above (see Figure 5 in Patton et al., 2013). In those studies, the motor pattern was labelled rectal motor complexes (RMCs), or period rectal motor activity (PRMA). Although negative or contradictory findings have been reported (Auwerda et al., 2001; Hagger et al., 2002), most 24 h studies have reported that this rectal activity was more frequent at night, compared to day (Kumar et al., 1989; Orkin et al., 1989; Ronholt et al., 1999; Rao et al., 2001a; Rao et al., 2001b; Rao et al., 2004). It was speculated that the increased nocturnal presence may help to prevent rectal filling while sleeping; a concept built upon with high-resolution manometry studies, which have now provided evidence for this rhythmic cyclic motor pattern acting as a rectosigmoid brake (Lin et al., 2017a; Lin et al., 2017b; Heitmann et al., 2022).

Compatible with the manometry data, an electromyographic (EMG) study of human colonic smooth muscle electrical behaviour distinguished long and short burst of spiking activity (Frexinos et al., 1985). However, short spike bursts were relatively constant, lacking daily rhythmicity, while long spike bursts were significantly more abundant during the day (Frexinos et al., 1985). In addition, total colonic pressure is reported to be lowest during the night, allowing accommodation of greater intraluminal volumes (Steadman et al., 1991). Indeed, colonic manometry combined with electroencephalography to monitor sleep stages revealed an inverse relationship between total colonic pressure and sleep depth (Furukawa et al., 1994).

Taken together, the available data suggest the human colon and rectum show complementary daily rhythmicity favouring increased diurnal motility in colon and nocturnal motility in the recto-sigmoid region. Food intake promptly enhances colonic motility but does not appear to fully account for daily rhythmicity, nor does ambulation. We speculate the daily rhythms in human colonic and rectal motor activity represent true circadian rhythms but this remains to be shown in temporally-isolated subjects.

In diurnal animals, available evidence shows similar daily rhythmicity to humans; total colonic contractility measured by pressure transducers in pigs was also significantly greater in the day compared to night time (Crowell et al., 1992). Colonic high amplitude propagating contractions in dogs, as measured by force transducers *in vivo*, were significantly more prominent in the early day period compared to other periods (Hirabayashi et al., 2009). In the chicken, EMG analysis of caecal and colonic smooth muscle firing activity revealed that periodic bursts of spikes that underlie contractility were relatively quiescent at night, compared to their frequency during the day (Rodriguez-Sinovas et al., 1994).

Colonic motor behaviour, *in vivo*, has also been assessed in nocturnal animals such as mice (Hoogerwerf et al., 2010), rats (Du et al., 1987; Gálvez-Robleño et al., 2022) and the house musk shrew, *Suncus murinus* (Kobayashi et al., 2022). In the house musk shrew, force transducers were used to detect ongoing contractility, including “giant migrating contractions” in the distal colon (GMCs) which were associated with defecation (Kobayashi et al., 2022). GMCs probably represent neurogenic peristalsis identified in more common experimental animals (Costa et al., 2013), and HAPCs in human colon (Spencer et al., 2016). The frequency of GMCs in the nocturnal house musk was almost 3 times higher in the night compared to the day period (Kobayashi et al., 2022). In mice, intracolonic pressure monitored *in vivo* showed a sustained elevation of basal pressure in the dark (active) period (Hoogerwerf et al., 2010), reminiscent of similar findings in humans (Steadman et al., 1991; Furukawa et al., 1994). Importantly, the daily oscillation in intracolonic pressure in mouse colon persisted under continuous dark conditions, consistent with circadian rhythmicity. In rats, colonic smooth muscle EMG recordings revealed periodic bursts of muscle action potentials. These spikes bursts were supressed during the day (inactive period), compared to the night (Du et al., 1987). Sympathetic preganglionic neurons to the prevertebral ganglia that in turn supply noradrenergic postganglionic neurons to the colon (Trudrung et al., 1994) are predominantly located in the intermediolateral column of the thoracolumbar spinal cord (Strack et al., 1988). Interestingly, thoracolumbar spinal cord ablation prevented the daily suppression of colonic spike burst activity (Du et al., 1987), suggesting thoracolumbar sympathetic drive may be required to suppress colonic motility during the inactive period. More recently, gastrointestinal transit was monitored by x-ray imaging after barium gavage in rats, revealing more rapid entry of content into the colon during the active period (Gálvez-Robleño et al., 2022). This effect was more pronounced in females than males (Gálvez-Robleño et al., 2022), similar to interactions between female sex and time of day in the rate of upper gastrointestinal transit in mice (Soni et al., 2019).

Recent data published in abstract form reports daily rhythmicity in the excitability of colonic myenteric neurons, *ex vivo* (Leembruggen et al., 2020); the enteric neural plexus underlying colonic neurogenic motility (Costa and Furness, 1976). Agonists to nicotinic, tachykinin, serotonin receptors and P2 purinoreceptors each evoked significantly greater intracellular calcium responses in the dark (active) period, compared to the light (inactive) period (Leembruggen et al., 2020), which may be consistent with observed differences in motility during these periods. The flat sheet *ex vivo* gut preparations used for this type of calcium imaging study are isolated from extrinsic neural, hormonal, and microbial inputs, thereby pointing to the role of intrinsic clock gene oscillations and their effectors in myenteric neurons as a potential mechanism for the observed differences in excitability between the active and inactive periods (Leembruggen et al., 2020).

#### Clock genes and colonic motility

Recent correlative analyses of genetic variation across multiple organs and cell types identify the colon as a major cross organ regulator of gene expression, showing more genes under rhythmic circadian control than any other organ analysed (Zhou et al., 2023). Most clock genes have been identified in the healthy colon and may be controlled by non-SCN peripheral influences. *Clock* and *Bmal1* mRNA are expressed in colonic epithelial cells and myenteric plexus (Hoogerwerf et al., 2007; Sládek et al., 2007), which are key coordinators of colonic function (Furness, 2012). The expression of both *Clock* and *Bmal1* peaks during the rest period and nadirs during the active period in humans, mice, and male rats (Hoogerwerf et al., 2007; Sládek et al., 2007; Sládek et al., 2012; Soták et al., 2013). Whilst males and females showed similar core clock gene phases, there were significantly more genes rhythmically expressed, with higher amplitudes, in female compared to male transverse colon (Talamanca et al., 2023). This suggests there are sex differences in the downstream output of the core circadian genes. *Per1/2*, *Cry1/2*, and *Rev-erb* are also expressed in the colon, showing an opposite phase to *Clock* and *Bmal1* where they peak during the active period and nadir during the inactive period in rats and mice (Hoogerwerf et al., 2007; Sládek et al., 2007; Sládek et al., 2012; Soták et al., 2013; Polidarová et al., 2014). ROR*α* has been identified in the colon, however, its research focus has been primarily on its involvement in colorectal cancers (Karasek et al., 2002; Winczyk et al., 2002). During constant darkness or light with *ad libitum* food access, rhythmic *Clock* expression in the male rat colon is lost whilst rhythms of *Bmal1*, *Per1/2*, and *Cry1/2* are maintained (Hoogerwerf et al., 2007; Sládek et al., 2007), suggesting dependence on an entraining light stimulus for rhythmic *Clock* expression. The persistence of *Bmal1*, *Per1/2*, and *Cry1/2* rhythmicity under constant light schedules is consistent with intrinsic circadian rhythmicity.

Feeding behaviour is rhythmic and under the influence of the SCN (Challet, 2019), thereby indirectly linking gut functions to light conditions. Bilateral SCN ablation in mice caused complete loss of faecal defecation rhythms, which may be attributed to loss of food intake rhythms (Malloy et al., 2012). Imposing rhythmicity of food intake by food restriction in SCN ablated mice restored defecation rhythms (Malloy et al., 2012), suggesting food intake is a strong influence. Indeed, reversed feeding times in rats results in reversal of colonic *Bmal1*, *Per1/2*, *Cry1/2*, and *Reverb* rhythmicity (Hoogerwerf et al., 2007; Sládek et al., 2007). However, the clock genes *Per2* and *Cry1* (but not *Clock*) in mouse distal colon continued to show daily rhythms following 24 h of constant darkness and fasting (Hoogerwerf et al., 2008). This shows that the rhythmicity of peripheral clocks in the colon withstands the removal of a more potent zeitgeber for the gut (food intake) than light, consistent with an intrinsic circadian rhythm.

Amongst core clock genes, only *Per1* and *Per2* have been investigated for a role in determining daily rhythms of colonic motility (Hoogerwerf et al., 2010). A *Per1*/*Per2* double gene knockout in mice (but not *Per1* or *Per2* knockout alone) abolished their daily rhythm of fecal pellet output, total colonic pressure and cholinergic agonist sensitivity in continuous dark conditions (120 h), leading to the conclusion that daily colonic motility rhythms are regulated by *Period* genes (Hoogerwerf et al., 2010). Whilst this conclusion may be correct, it has since been shown that the feeding behaviour of *Per1/Per2* double knockout mice becomes arrhythmic in constant darkness conditions (Adamovich et al., 2014), which provides an alternative explanation for the loss of colonic motor rhythms (Hoogerwerf et al., 2010). Indeed, only 48 h of an altered feeding schedule was required to alter colonic clock gene expression (Hoogerwerf et al., 2007). Imposed feeding rhythms or cell-specific knockouts may be able to rule out a role of arrhythmic feeding behaviour to bolster the conclusion that *Period* genes are responsible for circadian rhythms of colonic motility.

Beyond core clock genes, important neurotransmitters used by myenteric neurons have been reported to show daily rhythms. For example, a loss of daily colonic motor rhythms was observed in neuronal nitric oxide synthase (nNOS) knockout mice (Hoogerwerf, 2010) suggesting these rhythms are neuronally mediated. However, it is currently unknown how nNOS is linked to core circadian genes in the gut, if at all. Daily variation in mouse colonic *Calcb* gene expression has also been reported (Drokhlyansky et al., 2020; Leembruggen et al., 2020). This gene encodes the β-calcitonin gene-related peptide, which excites myenteric neurons (Palmer et al., 1986) and selectively expressed by mouse colonic intrinsic primary afferent neurons (Furness et al., 2004; Thompson et al., 2008; Hibberd et al., 2022c). This class of enteric neuron may be responsible for initiating excitation of enteric motor circuits to sensory stimuli (Kunze and Furness, 1999) and generating cyclic motor patterns (Hibberd et al., 2022b). Thus, variations in *Calcb* expression may contribute to daily rhythms in colonic motility.

#### Extrinsic neural control of motility

The colonic myenteric plexus is the principal coordinator of colonic motor behaviour (Costa and Furness, 1976), allowing the persistence of propulsive activities even in absence of central inputs (Bayliss and Starling, 1900). Nevertheless, the colon receives dense innervation from extrinsic noradrenergic sympathetic nerves (Tassicker et al., 1999; Olsson et al., 2006; Parker et al., 2022) which potently inhibits motility by supressing myenteric neurotransmission via action on presynaptic α_2_-receptors (Hirst and McKirdy, 1974; Stebbing et al., 2001) and actions on non-neural elements (Gillespie, 1962; Beani et al., 1969; Furness, 1969; Gulbransen et al., 2010; Kurahashi et al., 2020a; Kurahashi et al., 2020b; Zhang et al., 2022). Sympathetic outputs are under SCN control (Ueyama et al., 1999) and influence circadian rhythmicity of peripheral organs (Warren et al., 1994; Vujovic et al., 2008). Tyrosine hydroxylase activity, required for noradrenaline synthesis in sympathetic neurons, also shows circadian rhythmicity in the coeliac-superior mesenteric ganglia (Brusco et al., 1998); a major source of sympathetic innervation in the colon (Trudrung et al., 1994). Peripheral sympathetic nerve output may also be modulated by retinal light exposure (Niijima et al., 1992; Niijima et al., 1993; Mutoh et al., 2003; Ishida et al., 2005). Like other entraining factors, sympathetic influence on the colon may contribute to rhythmicity entrainment but is not essential, since rhythmic clock gene expression and fecal output patterns in mice persisted following sympathectomy but could be phase shifted by adrenergic receptor agonists (Malloy et al., 2012). On the other hand, an earlier study found sympathetic ablation abolished circadian fecal output patterns in rats, suggesting a more critical role (Du et al., 1987). In any case, the extrinsic sympathetic influence on colonic motility raises the possibility of circadian modulation of other colonic functions under sympathetic control, such as secretion and blood flow (Szurszewski and Linden, 2012). It is worth mentioning that gut epithelial cell proliferation shows circadian rhythmicity (Buchi et al., 1991; Marra et al., 1994; Scheving, 2000; Bjarnason and Jordan, 2002; Pácha and Sumová, 2013; Balounová et al., 2020) which is principally determined by feeding patterns (Yoshida et al., 2015) but are also modulated by sympathetic input (Tutton and Barkla, 1980; Kennedy et al., 1983; Tutton and Barkla, 1989). Parasympathetic vagal efferents are another potential source of extrinsic influence on the colon (Berthoud et al., 1991) that could impact circadian rhythmicity in motility, but few data are currently available. In mice, vagal pathways regulate clock gene expression in respiratory tissues (Bando et al., 2007), but were not required for the maintenance of clock gene rhythmicity in the stomach (Hoogerwerf et al., 2007).

#### Microbial products and circadian control of colonic function

Intraluminal products of microbial metabolism, particularly secondary bile acids and short chain fatty acids (SCFAs), have received attention as potential circadian entraining factors. Microbes and their metabolites are themselves subject to daily rhythms, highlighting a major potential source of variability in studies of the microbiome (Allaband et al., 2022). Partly driving these oscillations is rhythmic delivery of intraluminal content to the gut by feeding behaviour that is ultimately controlled by the SCN (Nagai et al., 1978) and clock gene oscillations (Turek et al., 2005). Gut microbial characteristics, including relative abundances, spatial organization and metabolism oscillate with feeding rhythmicity (Thaiss et al., 2014; Zarrinpar et al., 2014; Thaiss et al., 2016), modulating circadian profile of host peripheral gene transcription programs via direct microbe-epithelium interactions (Abreu, 2010; Wells et al., 2011; Mukherji et al., 2013; Clasen et al., 2023) and microbial metabolites such as polyamines, SCFAs and unconjugated bile acids (Leone et al., 2015; Govindarajan et al., 2016; Thaiss et al., 2016; Tahara et al., 2018). Specifically, the SCFAs evoked shifts in clock gene expression of multiple peripheral cell types (Leone et al., 2015; Tahara et al., 2018), including colonic epithelia (Desmet et al., 2021b). Yet, despite their coordinating influence, microbial entraining mechanisms may not be strictly necessary for peripheral core clock entrainment, since peripheral clock gene rhythmicity persisted following microbial ablation (Thaiss et al., 2016). Indeed, microbial circadian rhythmicity may depend on gut epithelial circadian clocks (Mukherji et al., 2013; Altaha et al., 2022; Heddes et al., 2022), although time-restricted feeding recapitulates features of normal microbial oscillation after core clock gene knockout (Thaiss et al., 2014; Segers et al., 2020).

Endogenous circadian rhythms have been present throughout evolution (Jabbur and Johnson, 2021), and the molecular clock used by Cyanobacteria is well characterised (Johnson et al., 2017). There is currently limited evidence for intrinsic circadian rhythms in non-photosynthetic bacteria (Eelderink-Chen et al., 2021) but the field of prokaryotic chronobiology has been described as young compared to the study of eukaryotic circadian systems (Johnson et al., 2017), largely leaving open the question whether gut microbes have their own oscillators. At least one bacterial species in the human gut microbiome has been identified that shows entrainable, temperature-compensating circadian oscillations, *in vitro* (Paulose and Cassone, 2016; Paulose et al., 2016; Paulose et al., 2019).

SCFAs arise from microbial metabolism of undigested carbohydrates; they have been identified in the gut of amphibians, birds, reptiles, fish, and mammals, including humans (McNeil, 1984; Pryor and Bjorndal, 2005; Blaak et al., 2020). In mammals, most SCFAs are produced in the caecum and colon (den Besten et al., 2013), with concentrations showing daily oscillation. In mice and rats fed *ad libitum*, most reports of caecal and blood SCFAs show peak concentrations around the early to mid-active period (Tahara et al., 2018; Segers et al., 2020; Han et al., 2021; Ding et al., 2022), preceding a colonic peak from the late active to mid inactive period (Henning and Hird, 1972; Yajima and Sakata, 1992; Segers et al., 2019; Desmet et al., 2021a; Desmet et al., 2021b). Core clock gene *Bmal1* knockout in mice disrupted feeding patterns, microbial rhythmicity (Liang et al., 2015), and circadian SCFA fluctuations (Segers et al., 2019). Interestingly, sleep duration correlated with SCFA production in humans (Shimizu et al., 2023), who also show daily fluctuations in circulating SCFAs, peaking in the latter half of the day, after lunch and dinner (Wolever et al., 1997; Swanson et al., 2020; Brignardello et al., 2022). Peak colonic concentrations, particularly in the distal regions are presumed to be somewhat later.

Aside a potential role in entraining circadian signalling, the question arises whether cycling colonic SCFA levels may more directly exert regulatory effects on colonic functions, such as colonic motility. Reports of the acute colonic motor effects of single or multiple SCFAs range from predominantly inhibitory (Squires et al., 1992; Ono et al., 2004; Dass et al., 2007; West et al., 2017), mixed (Cherbut et al., 1998; Mitsui et al., 2005a; Hurst et al., 2014; Shaidullov et al., 2021), excitatory (Yajima, 1985; McManus et al., 2002; Fukumoto et al., 2003; Rondeau et al., 2003; Mitsui et al., 2005b; Grider and Piland, 2007; Tan et al., 2020), or without detectable effects (Flourie et al., 1989; Jouët et al., 2013; Vincent et al., 2018). Similarly, chronic SCFA elevation by various methods have shown inhibitory effects on colonic transit and contractility (Bardon and Fioramonti, 1983; Bajka et al., 2010; Patten et al., 2015; Yuan et al., 2020), or increased transit and contractility (Soret et al., 2010; Suply et al., 2012). Taking these and other considerations (Sakata, 2019) into account, it is difficult to determine how SCFA rhythmicity may affect the circadian cycle of colonic motility, if at all. To this end, Segers et al. (2019) quantified SCFA-mediated inhibition of nerve evoked contractility in proximal and distal colonic strips across the circadian cycle. Maximal and minimal inhibition occurred in the inactive and active periods, respectively, paralleling oscillation in expression of free fatty acid receptors 2 and 3 (Segers et al., 2019). This would suggest SCFA oscillation may indeed support inhibition of colonic motility in the inactive period. However, it will be important to show whether propulsion is also affected, as studies of acute SCFA application have occasionally identified inhibitory effects on contractility whilst facilitating colonic propulsive behaviour (Cherbut et al., 1998; Tan et al., 2020; Shaidullov et al., 2021).

Finally, it may be speculated that colonic SCFAs exert long range motility effects. Since the enteroendocrine cells and neural circuits underlying the ileal brake also exist in colon (Szurszewski and Linden, 2012; Hibberd T. et al., 2022; Holst et al., 2022; Zhang et al., 2022), an untested possibility is that SCFAs contribute to glucagon like peptide 1 (GLP-1) and peptide tyrosine tyrosine (PYY) release from colonic enteroendocrine cells (Freeland and Wolever, 2010; Psichas et al., 2015; Christiansen et al., 2018; Larraufie et al., 2018), supporting upper gastrointestinal inhibition at the endogenous SCFA daily peak via an ileal brake mechanism (Van Citters and Lin, 2006; Zhang et al., 2022). Compatible with this, intracolonic infusion of exogenous SCFAs suppressed gastric tone in humans, coinciding with elevated plasma PYY but not GLP-1 (Ropert et al., 1996).

Primary bile acids are delivered to the small intestine for nutrient digestion and can be transformed by intraluminal bacteria that express bile salt hydrolase to form secondary bile acids. These microbially-modified bile acids show daily rhythmicity in blood (Setchell et al., 1982; Steiner et al., 2011; Zhang et al., 2011; Eggink et al., 2017; Al-Khaifi et al., 2018) and faecal concentrations (Cui et al., 2022a; Altaha et al., 2022; Cui et al., 2022b), and may modify peripheral clock gene expression in the ileum, colon and liver (Govindarajan et al., 2016). Like SCFAs, secondary bile acids can exert direct effects on colonic motility (Alemi et al., 2013). Interestingly, circadian disruption evoked *de novo* circadian rhythmicity in bile acid receptor expression (Desmet et al., 2023).

#### Colonic motility and disruptions of colon rhythms in IBS and UC

Irritable bowel syndrome (IBS) is a functional gastrointestinal disorder characterised by recurrent abdominal pain and altered bowel habits: (constipation, diarrhea, or both; Moayyedi et al., 2017). More than 90% of patients experience abdominal pain; the symptoms that most severely disrupts quality of life (Cain et al., 2006; American Gastroenterological Association, 2015; Mearin et al., 2016). Gut symptoms of IBS and functional dyspepsia are significantly exacerbated by disruptions of circadian rhythms (Kim et al., 2013; Fowler et al., 2022). Circadian disruptions commonly occur through shift work, or work outside the normal 9a.m.-5p.m. hours. Shift work is strongly associated with an increased prevalence of IBS-related symptoms such as constipation or diarrhea, bloating, gas, and abdominal pain (Wells et al., 2012; Kim et al., 2013; Hyun et al., 2019; Rahimimoghadam et al., 2020; Roman et al., 2023), and alterations in the composition of the gut microbiome (Mortaş et al., 2020). In constipation-related IBS (IBS-C), the frequency of high-amplitude propagating colon contractions in patients are decreased over a 24-period (Bassotti et al., 2003). Conversely, in diarrhoea-related IBS (IBS-D) patients, the frequency of high-amplitude propagated contractions were higher during the active period compared to controls (Clemens et al., 2003). Simulated shift work in mice led to increased colon motility and permeability (Summa et al., 2013; Tran et al., 2021), and decreased apical junction complexes (Tran et al., 2021); factors which likely contribute to IBS-D.

Inflammatory bowel diseases, including UC, are chronic relapsing gastrointestinal disorders with increasing prevalence worldwide (Ng et al., 2017). Most patients with UC experience abdominal pain throughout their disease, profoundly impacting their quality of life (Zeitz et al., 2016). The severity of UC, characterised by inflammation and development of ulcers in the colon, is exacerbated by circadian disruptions. In humans, sleep disruptions worsened UC symptoms with increased colon permeability and pro-inflammatory cytokines (Sobolewska-Włodarczyk et al., 2020; Swanson et al., 2021). Animal studies suggest the increased severity of UC associated with circadian disturbances is likely due to impaired recovery. Clock controlled genes are implicated by observations that deletion of *Bmal1* in dextran sulfate sodium (DSS)-induced colitis mice delayed colon epithelium regeneration via disruptions to rhythms of cell proliferation (Taleb et al., 2021) suggesting *Bmal1* is necessary for UC recovery. Further, jetlag-induced circadian disruptions in DSS-induced colitis mice aggravated colitis, disrupted rhythms of *Clock* and *Bmal1* expression, and reduced *Per2* expression. Decreased *Per2* expression was associated with decreased adenosine triphosphate and cell proliferation in the colonic epithelium via circadian modification of dynamin-related protein 1, which mediates mitochondrial fission (Chen et al., 2022).

### Circadian rhythms of colonic absorption, permeability, and hormone secretion

#### Absorption

The human colon contributes to body water balance by reabsorbing 1.5–2 L of daily fluid inputs, which represents ∼20% of the total fluid intake of the gut (Barrett and Keely, 2022). One of the primary ways this is achieved is via electrogenic import of sodium ions through epithelial sodium channels (ENaC) located on the apical membrane of mucosal cells (Kunzelmann and Mall, 2002). Daily rhythmicity in electrical potential difference across colonic epithelium, reflecting changes in electrogenic absorption, was reported in rabbit colon and rectum with peak absorption in the dark period (Clauss, 1984; Clauss et al., 1988). Rabbits produce two types of faeces, hard and soft, which are excreted in the dark (active) and light (inactive) periods, respectively (Jilge, 1974). The latter are reingested during the light period (Jilge and Hudson, 2001), recovering nutrients made available by hindgut fermentation, including SCFAs (Henning and Hird, 1972; Vernay et al., 1984; Vernay, 1989). The least colonic reabsorption of sodium and water in the light period coincides with soft faeces production in rabbits. In contrast, mice and rats have more uniform faeces than rabbits but also show daily rhythms of colonic and rectal sodium absorption via amiloride-sensitive ENaC (Wang et al., 2000; Wang et al., 2004; Wang et al., 2010; Frateschi et al., 2012; Malsure et al., 2014). In mice and rats, the night (active) period is the peak period for both sodium reabsorption and defecation.

In addition to ENaC-mediated electrogenic transport, electroneutral absorption via Na^+^/H^+^ exchangers may have circadian rhythmicity as transcription of Na^+^/H^+^ hydrogen exchanger 3 (*Nhe3*) in rat colonic epithelium showed circadian rhythmicity under constant lighting conditions, peaking in the night (active) period (Sládek et al., 2007; Soták et al., 2011), paralleling the daily cycle of electrogenic transport via ENaC.

#### Corticosteroid influences on absorption

The early studies of colonic absorption identified the parallel rhythmic oscillations in corticosteroids as possible underlying mechanism for daily rhythms of absorption (Clauss, 1984; Clauss et al., 1988). Indeed, adrenalectomy blunted circadian rhythmicity in *Nhe3* in intestinal epithelia (Vagnerová et al., 2019) and clock gene rhythmicity in colonic epithelia, which could be restored by exogenous gluococorticoids (Polidarová et al., 2017). Mineralocorticoids are also candidate entrainers of colonic absorption as aldosterone may entrain renal ENaC via regulation of *Per1* (Gumz et al., 2009).

#### Permeability

Colonic permeability has been positively correlated with stool frequency in rats (Hou et al., 2019). Compatible with this, colonic permeability is reported to have a daily rhythm in mice, peaking in the night (active) phase: the period of greatest faecal pellet output (Oh-oka et al., 2014). Epithelial tight junctions are the main regulators of colonic permeability (Lee, 2015). Some evidence suggests tight junction proteins such as occludins and claudins, may be expressed with daily rhythmicity in the colon, putatively controlled by CLOCK-BMAL1 (Oh-oka et al., 2014). Colonic permeability is inversely associated with the expression of the occludin and claudin proteins. Colonic epithelial occludin mRNA expression peaked during the day (inactive) period and nadirs during the night (active) period in mice (Summa et al., 2013; Oh-oka et al., 2014; Desmet et al., 2021b). Evidence is currently mixed as to whether the same pattern occurs with colonic epithelial *Claudin-1* mRNA expression (Oh-oka et al., 2014; Desmet et al., 2021b) and *Bmal1* knockout did not affect colonic *Claudin-1* mRNA expression in a recent study (Taleb et al., 2022).

#### GLP-1 secretion

Epithelial L-cells secrete the hormone glucagon-like peptide 1 (GLP-1) in response to luminal nutrients such as glucose, potentiating pancreatic glucose-evoked insulin secretion while inhibiting glucagon secretion (Drucker, 2018; Holst, 2022) and contribute to the so called “ileal-brake” (Read et al., 1984; Spiller et al., 1984) to acutely inhibit appetite (Flint et al., 1998; Giralt and Vergara, 1998; 1999; Zhang et al., 2022). L-cells occur in large numbers in the distal small intestine (Knudsen et al., 1975; Eissele et al., 1992) where their physiological effects are best characterised. Interestingly, their density increases along the colon and rectum where the role of GLP-1 is less understood (Holst et al., 2022) and are more likely activated by bile acids (Christiansen et al., 2019) rather than nutrients like glucose that are absorbed in the more proximal gut.

A daily rhythmicity of GLP-1 secretion was suggested by the observation that identical meals consumed at different times evoked significantly different plasma GLP-1 responses in humans, favouring higher GLP-1 secretion in the morning, compared to evening (Lindgren et al., 2009). A circadian rhythmicity of GLP-1 secretion was confirmed in rats (Gil-Lozano et al., 2014) and mice (Biancolin et al., 2020; Desmet et al., 2021b), depending on circadian rhythmicity of the BMAL1-controlled SNARE regulatory protein, secretagogin (Biancolin et al., 2020; Biancolin et al., 2022). Interestingly, GLP-1 secretion rhythmicity may not depend on entrainment by glucocorticoid rhythms (Gil-Lozano et al., 2016). However, GLP-1 secretion and L-cell core clock gene rhythms were deranged by high fat diets and microbial ablation, pointing to a critical role for the microbiome in maintaining GLP-1 secretion rhythmicity (Gil-Lozano et al., 2016; Martchenko et al., 2018; Martchenko et al., 2020).

### Circadian rhythm of colonic afferents and pain

Daily rhythmicity in pain perception in humans is commonly reported, with peak and nadir timing varying across sensory modalities and pathophysiological conditions (Aviram et al., 2015; Daguet et al., 2022; Mun et al., 2022). The first order neurons involved in sensory signalling from the colon are vagal and spinal afferents. In other gastrointestinal organs such as the stomach, mucosal and tension receptors of the vagal nerve have a circadian rhythm in mechanosensitivity, inversely proportionate to food intake (Page, 2021). Their excitability is higher at the onset of the active-compared to inactive period (Kentish et al., 2013). Currently no studies have investigated the circadian rhythm modulation of sensory vagal fibres that innervate the proximal or distal colon. However, recent work has identified that vagal afferent signalling to second order neurons in the nucleus tractus solitarius (NTS) also shows circadian variability that favours throughput of afferent-driven signalling during the active period, and passive spontaneous firing during the inactive period (Ragozzino et al., 2023). It remains to be determined whether similar mechanisms govern circadian variation of signalling efficacy to the CNS in spinal afferent pathways.

Colonic spinal afferents and their function have been reviewed extensively elsewhere (Brierley et al., 2018). In brief, colonic afferents send mechanical and chemical signals about the colon (e.g., luminal contents and wall stretch) to the spinal cord via the lumbar splanchnic and sacral pelvic nerves. These afferents have been classified into five major types, muscular, mucosal, muscular-mucosal, vascular, and silent (Brierley et al., 2018). Surprisingly, circadian rhythms of colonic afferents have, to date, not been directly investigated. Interestingly, bladder afferents derive from lumbar splanchnic and sacral pelvic nerves like the afferent supply to the distal colon and show strong time-of-day regulation of sensitivity, raising the possibility similar variations occur in colon. At least 3 classes of bladder afferents (stretch-insensitive mucosal and stretch-sensitive low and high threshold muscular-mucosal afferents) demonstrated significantly increased sensitivity to mechanical stimuli like stroking and stretch during the active-, compared to the inactive period, suggesting strong circadian regulation of spinal sensory neuron excitability (Christie and Zagorodnyuk, 2021; Ramsay and Zagorodnyuk, 2023).

In the distal colon, potential circadian regulation of colonic afferents could be inferred through measurements of visceromotor responses (VMRs), that can be assessed by recording abdominal EMG activity, evoked by colonic distension. Distension of hollow visceral organs evoked VMRs that may serve as an indirect indication of visceral afferent activity, and, at noxious distensions (>40 mmHg) VMRs are used as surrogate measure of pain (Ness and Gebhart, 1988; Ness and Elhefni, 2004; Zagorodnyuk et al., 2011; Kyloh et al., 2022). An early study reported that VMRs evoked by colorectal distension in rats exhibits a daily rhythm with significant increase in the response seen in active period (at night) (Gschossmann et al., 2001). However, a more recent study reported that distension-evoked VMRs in rats do not exhibit a daily rhythm (Botschuijver et al., 2016). The reason for this conflicting information between studies is not clear but may involve different distension methods (volume versus isobaric), conscious freely moving versus restrained animals, and/or differences in the strains of rats used. Compatible with the idea that visceral afferent sensitivity and signalling efficacy to the CNS may be enhanced during the active-compared to inactive period, human data indicates perception thresholds to rectal distension stimuli for urge and pain was lower in the morning than evening (Enck et al., 2009). Interestingly, daily variations in sensory signalling may differ by region and sensory modality; peak visceral pain sensitivities in the active period differs to those for cutaneous thermal and mechanical pain and in conditions like neuropathic pain and cluster headache which peak during the inactive period (Mun et al., 2022).

### Melatonin

Melatonin arises from multiple sources, of which the best known is nocturnally generated pineal melatonin. However, extra-pineal melatonin is a far greater source of melatonin in the body, much of which may be generated in mitochondria where it controls oxidative processes and which may represent its original site of synthesis in evolution (for review, see Tan et al., 2013; Zimmerman and Reiter, 2019; Tan et al., 2023). In the gut, melatonin is predominantly contained in the epithelial cells along the whole gastrointestinal tract (Bubenik et al., 1977; Bubenik, 1980; Holloway et al., 1980; Lee and Pang, 1993; Poon et al., 1996; Söderquist et al., 2015). Like serotonin (Gershon, 2022), more of the body’s melatonin is synthesized in the gut than in the brain (Huether, 1993). Both melatonin and serotonin released from mucosa give rise locally to micromolar concentrations in mouse ileum and colon (Bertrand et al., 2010; Diss et al., 2013).

#### Melatonin effects on gut smooth muscle

Melatonin is both water and lipid-soluble, so it can penetrate the cell membrane and act on intracellular receptors of the RORα family and/or directly on intracellular proteins including Ca^2+^ binding protein, calmodulin and Ca^2+^/calmodulin-dependent kinase II (CaMKII) (Landau and Zisapei, 2007; Hardeland et al., 2011; Han et al., 2012). Melatonin is capable of inhibiting smooth muscle of urogenital organs including myometrium and detrusor muscle: these direct effects likely due to its ability to inhibit Ca^2+^ channels and Ca/MKII system (Ouyang and Vogel, 1998; Ayar et al., 2001; Han et al., 2012).

Melatonin may have two different effects on the vascular smooth muscle, with vasoconstriction mediated via MT1 and vasodilation–via MT2 (Harlow and Weekley, 1986). In dispersed gastric smooth muscle cells, melatonin-evoked contraction was mediated by MT1 activation of G_q_ to stimulate phosphoinositide hydrolysis and increased cytosolic Ca^2+^ (Ahmed et al., 2013). In small gut segments, melatonin decreased rat small intestine and colon contractility, whereas it evoked contraction of guinea pig proximal colon (Harlow and Weekley, 1986; Lucchelli et al., 1997). Melatonin’s inhibitory effects on rat ileal smooth muscle may be mediated by Ca^2+^ activated K^+^ channels (Reyes-Vázquez et al., 1997). Smooth muscle responses to melatonin in the studies by Lucchelli et al. (1997) and Reyes-Vázquez et al. (1997) were not significantly affected by neuronal blockade, suggesting enteric neurons were not involved. Taken together, melatonin has potential to directly affect colonic smooth muscle function, but its importance under normal physiological conditions is not characterised.

#### Melatonin effects on the enteric neurons

In enteric neurons, MT1 receptor immunofluorescence was weak or undetectable in human colonic submucous and myenteric plexus, but MT2 receptor immunoreactivity was generally stronger, ranging from weak to strong in both plexuses (Söderquist et al., 2015). *Mtnr1a* mRNA was also reported in rat small intestine myenteric neurons (Soták et al., 2006). Electrophysiologically, exogenous melatonin did not affect membrane potential or input resistance, but inhibited nicotinic synaptic input in guinea pig ileum submucous neurons (Barajas-López et al., 1996). In mouse colon, an inhibitory action of melatonin on neuronal NOS was inferred by its reduction of the slow (nitric oxide-mediated) (Shuttleworth et al., 1997; Kuriyama et al., 1998) component of the inhibitory junction potential (Storr et al., 2002). Whether these actions of exogenous melatonin relate to any endogenous role, or the circadian regulation of colonic functions remains to be established.

#### Melatonin and gut motility

Melatonin is released into circulation by the pineal gland during the dark and is hormonal regulator of circadian rhythms. There is some evidence of pineal melatonin involvement in regulation of the interdigestive migrating motor complex (MMC; Szurszewski, 1969) in rats (Bonouali-Pellissier, 1994). Pineal or exogenous melatonin does not affect clock gene expression in rat or mouse colonic epithelial cells (Polidarová et al., 2017), suggesting melatonin plays no role in entraining these peripheral clocks. Melatonin is produced peripherally (Huether et al., 1992; Huether, 1993) in a non-circadian manner (Bubenik, 2002) by the gut enterochromaffin cells in response to food intake, with melatonin levels sharply rising after a meal (Bron and Furness, 2009; Duboc et al., 2020). Exogenous melatonin can modulate colonic transit, and this may be dose dependent. One study has demonstrated that 3 mg of melatonin daily increases colon transit time in healthy humans (Lu et al., 2009). Another study in rats reported that low doses of melatonin (10 μg/kg) increased colonic transit whilst high doses (1 mg/kg) decreased it (Drago et al., 2002), suggesting a potential biphasic effect, which is often seen for G-protein coupled receptors. The underlying mechanisms of melatonin action on colonic motility are not known. In *in vivo* studies of the small intestine, nonselective MT1 and MT2 melatonin receptor antagonist, S-22153 suppresses nocturnal variations in interdigestive MMC frequency in the rat small intestine (Merle et al., 2000). This may suggest an involvement of melatonin in physiological regulation in the pre- and postprandial changes of intestinal motility (Merle et al., 2000). Melatonin in pharmacological doses (1 mg/kg) increased frequency of MMC by reducing the duration of irregular spiking activity and of the quiescent period (Merle et al., 2000).

#### Melatonin in the treatment of IBS and UC

Melatonin has potential as a therapeutic for the treatment of IBS and UC symptoms, although reports are conflicting. It has been shown that melatonin (3 mg) improves abdominal pain associated with both IBS-C and IBS-D (Song et al., 2005). However, it is also reported that melatonin (3 mg) improves abdominal pain in only IBS-C and not IBS-D (Chojnacki et al., 2013). Other studies also indicated that melatonin (3 mg) improved abdominal pain, however, the type of IBS was not specified (Saha et al., 2007). Similarly, the effect of melatonin on stool frequency and colonic transit in IBS is conflicting. It has been shown that melatonin (3 mg) only improves stool frequency and colonic transit in IBS-C patients (Chojnacki et al., 2013; Mishchuk et al., 2019). However, it is also reported that melatonin has no effect on stool frequency and colonic transit in IBC-D and IBC-C patients compared with placebo (Lu et al., 2009). It should be noted that other, greater affinity, MT1 and MT2 agonists, such as agomelatine, have been studied for their potential in the treatment of IBS-D. Agomelatine (25 mg) significantly improved overall symptoms in IBS-D patients (Balakina et al., 2014). However, agomelatine is also a 5-HT_2C_ and 5-HT_2B_ receptor antagonist (Guardiola-Lemaitre et al., 2014) which suggests agomelatine may influence colonic motility acting on 5-HT receptors.

As previously mentioned, disruptions to circadian rhythms can exacerbate UC signs and pathology. In UC-circadian disrupted mice, treatment with melatonin reduced the signs and severity of inflammation in the colon (Park et al., 2015; Liu and Wang, 2019) which was abolished by the non-specific MT1 and MT2 antagonist luzindole (Liu and Wang, 2019). Similar effects of melatonin are also seen in UC mice without circadian disruptions (Trivedi and Jena, 2013). It has been speculated that patients with UC may have increased synthesis of melatonin in the colonic mucosa (Vaccaro et al., 2023). It is likely that in the treatment of UC, melatonin exhibits a protective, anti-oxidative effect on the colonic mucosa.

## Conclusion

A wide array of colonic functions shows circadian rhythmicity optimized to the period of food intake. Disruptions of these rhythms can cause organ disorders or exacerbate pre-existing ones. Multiple neural, hormonal and intraluminal mechanisms may contribute to the entrainment of circadian variation in colonic functions, but their full details remain to be elucidated. Gut melatonin, in contrast with pineal melatonin, may be principally arrhythmic in function but nevertheless may have therapeutic potential in its exogenous application for treatment of gut disorders that are exacerbated by circadian disruption.
